# Supplementary material for: Association of Radiation Dose to the Amygdala–Orbitofrontal Network with Emotion Recognition Task Performance in Patients with Low-Grade and Benign Brain Tumors
Source: Cancers (Basel). 2023 Nov 23;15(23):5544. doi: 10.3390/cancers15235544 (PMC10705220; doi:10.3390/cancers15235544)
Supplement: Supplementary file 1 [file cancers-15-05544-s001.zip › cancers-2674709-supplementary.pdf]

cancers

MDPI

Association of Radiation Dose to the Amygdala-Orbitorfrontal Network with Emotion Recognition Task Performance in Patients with Low-Grade and Benign Brain Tumors

Sara J Hardy, Alan Finkelstein, Michael T Milano, Giovanni Schifitto, Hongying Sun, Koren Holley, Ken Usuki, Miriam T. Weber, Dandan Zhang, Christopher L. Seplaki and Michelle Janelsins

Table S1. Additional demographic information for participants.

Variable	N (%)
Education level	
Vocational/Technical school	2 (4%)
High school graduate or GED	11 (22%)
Associates degree or some college	12 (24%)
Bachelor's degree	14 (27%)
Masters or higher degree	12 (24%)
Work status	
Not currently working	13 (25%)
Currently working	22 (43%)
Retired	16 (31%)
Smoking status	
Current or prior smoker	20 (39%)
Never smoker	31 (61%)

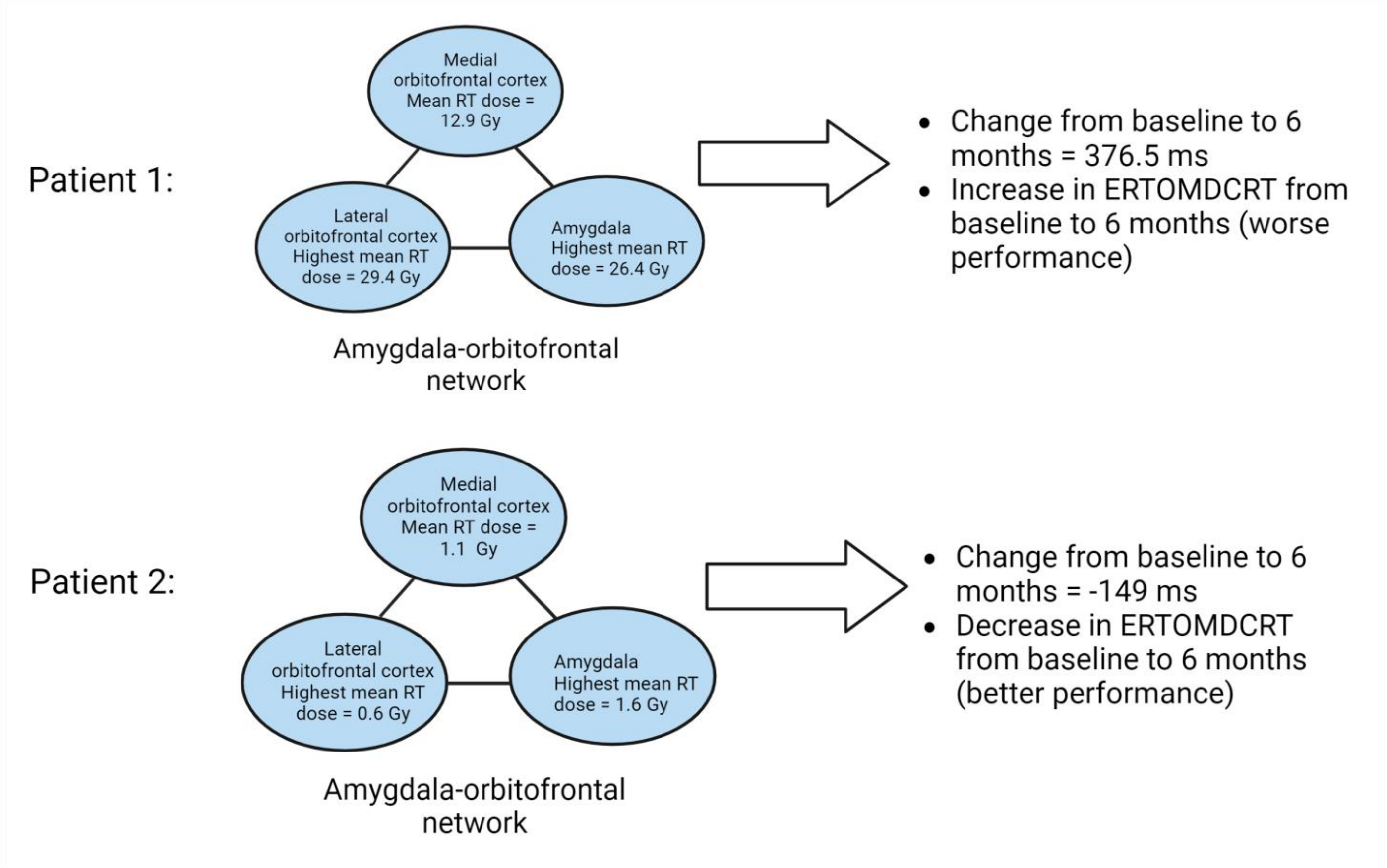

Figure S1. Radiation doses to the amygdala-orbitofrontal network and changes on the ERT-OMDCRT for two representative participants in the cohort.
